# NO-donors induce cross talk between cGMP and cAMP in signalling to human atrial L-type Ca^2+^ current

**DOI:** 10.1186/1471-2210-11-S1-P55

**Published:** 2011-08-01

**Authors:** Nadiia Rozmaritsa, Torsten Christ, Erich Wettwer, Ursula Ravens

**Affiliations:** 1 Department of Pharmacology and Toxicology, Dresden University of Technology, Dresden, 01307, Germany

## Background

Cardiac NO-activated pathways are discussed to involve cross-talk between cGMP and cAMP signalling [1,2]. Here we have investigated the signalling pathways relating to NO-donor S-nitroso-N-acetylpenicillamine (SNAP) modulation of L-type Ca^2+^ current (I_Ca,L_) in human right atrial cardiomyocytes.

## Material and methods

Experiments were performed on human biopsy tissue from 62 patients in sinus rhythm. I_Ca,L_ was measured with whole-cell voltage-clamp technique.

## Results

**Figure 1:**
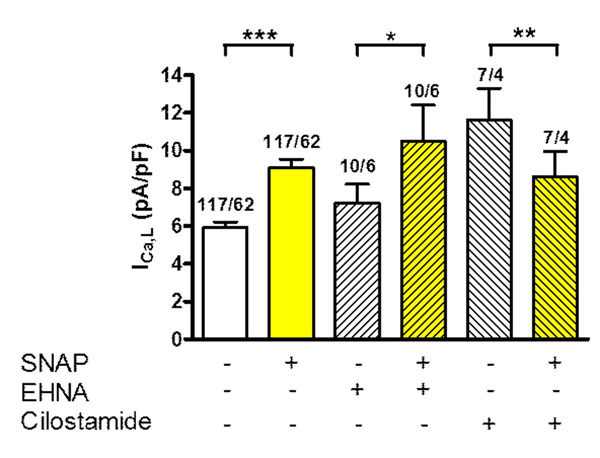
Effect of SNAP (100µM), PDE2 inhibitor (EHNA, 10µM) and PDE3 inhibitor (Cilostamide, 1µM) on I_Ca,L_. SNAP effect in the presence of cilostamide.

Application of SNAP (100µM) increased basal I_Ca,L_ from 5.93±0.23 pA/pF to 9.10±0.45pA/pF (p<0.001, n/N=117/62). The effect was abolished by inhibition of soluble guanylate cyclase (sGC) with ODQ (30µM), suggesting involvement of cGMP. Stimulator of sGC (BAY 41-2272, 10nM–10µM) also increased I_Ca,L_ and this effect was potentiated in the presence of SNAP. Direct activation of protein kinase G (PKG) with 8-Br-cGMP (100 µM, intracellular application) increased basal I_Ca,L_. However, not only cGMP but also cAMP was involved, because, the effect of SNAP on I_Ca,L_ was prevented with the protein kinase A blocker (Rp-8-Br-cAMP 1 mM, intracellular). Thus, cGMP may activate I_Ca,L_ via direct activation of PKG and indirect activation of PKA at the same time. It is known, that cAMP-mediated activation of PKA is regulated by cGMP via modulation of phosphodiesterases (PDEs). The selective PDE2 inhibitor EHNA (10µM) did not affect basal or SNAP-stimulated I_Ca,L_, therefore PDE2 does not regulate basal cAMP level. In contrast, PDE3 inhibition with cilostamide (1µM) increased basal I_Ca,L_, suggesting that PDE3 is involved in basal cAMP level regulation. Interestingly, the cilostamide-induced increase in I_Ca,L_ is blunted upon addition of SNAP, most probably via activation of PDE2 by SNAP-mediated cGMP increase (Figure 1). Similarly, SNAP blunted enhancement of I_Ca,L_ by PKA activation with isoprenalin (1µM; 18.07 ± 1.12 pA/pF vs 23.06 ± 1.36 pA/pF, p<0.001, n/N=21-39/18), however, this effect was prevented by PDE2 inhibition with EHNA.

## Conclusion

We conclude that in human atrial cardiomyocytes NO-donors stimulate production of cGMP with further cross-talk to cAMP via PDE2 and PDE3.
